# Associations of lifestyle and diet with the risk of nasopharyngeal carcinoma in Singapore: a case–control study

**DOI:** 10.1186/s40880-016-0174-3

**Published:** 2017-01-07

**Authors:** Sook Kwin Yong, Tam Cam Ha, Ming Chert Richard Yeo, Valerie Gaborieau, James D. McKay, Joseph Wee

**Affiliations:** 1 Division of Clinical Trials and Epidemiological Sciences, National Cancer Centre Singapore, 11 Hospital Drive, Singapore, 169610 Singapore; 2 Medical Education, Research & Evaluation Department (MERE), Duke-NUS Medical School, 8 College Road, Singapore, 169857 Singapore; 3 Division of Radiation Oncology, National Cancer Centre Singapore, 11 Hospital Drive, Singapore, 169610 Singapore; 4 Genetic Cancer Susceptibility Group, International Agency for Research on Cancer, World Health Organization, 150 Cours Albert Thomas, 69372 Lyon Cedex 08, France

**Keywords:** Nasopharyngeal carcinoma, Diet, Smoking, Alcohol drinking, Case–control study, Preserved food

## Abstract

**Background:**

Nasopharyngeal carcinoma (NPC) is a commonly diagnosed cancer in Southeast Asia. Many studies have examined the risk factors for NPC, yet the roles of some risk factors remain inconclusive. The purpose of this study was to examine associations between modifiable lifestyle factors and the risk of NPC in the Singaporean population.

**Methods:**

We conducted a case–control study in Singapore with 300 patients and 310 controls who were recruited between 2008 and 2012. Each control was selected and individually matched to each patient based on sex, ethnicity, and age (±5 years). A total of 290 pairs of cases and controls were matched successfully. We examined lifestyle factors such as tobacco smoking, alcohol drinking, various salted and preserved food consumption, and weaning practices.

**Results:**

After adjusting for covariates, multivariate analysis showed that those participants who were current smokers and had ever smoked tobacco had a higher risk of NPC than participants who had never smoked, with odds ratios (ORs) of 4.50 (95% confidence interval [CI] 2.58–7.86; *P* < 0.001) and 2.52 (95% CI 1.54–4.12; *P* < 0.001), respectively. Those who consumed salted vegetables at least once a week also showed a significantly increased risk of NPC than those who never or rarely consumed salted vegetables, with an OR of 4.18 (95% CI 1.69–10.38; *P* = 0.002).

**Conclusion:**

Smoking (currently and ever-smoked) and consuming salted vegetables once a week or more were lifestyle risk factors for NPC, and changes of these factors for the better may reduce the risk of NPC.

## Background

Nasopharyngeal carcinoma (NPC) is a commonly diagnosed cancer in Southeast Asia. Indeed, NPC is endemic in China (especially South China, Hong Kong, and Taiwan) and Southeast Asia countries such as Malaysia and the Philippines. For example, in 2010, South China was reported to have an age-standardized rate (ASR) of incidence of 2.44 per 100,000 people and an ASR of mortality of 1.18 per 100,000 people [1]; the Philippines was reported to have an ASR of incidence of 1.79 per 100,000 people [2]. In Singapore, NPC is most commonly diagnosed in men; in 2012, the ASR of incidence of NPC reported in men was 8.8 per 100,000 people [3].

Numerous studies have been conducted in Southeast Asia to identify risk factors related to the development of NPC [4–14]. Some of these studies suggested that NPC results from an interaction of genetic and environmental factors [7, 10, 11, 15]. Studies conducted in a Chinese population in Hong Kong, China found that over 90% of people younger than 35 years consumed salted fish during childhood and that they have continued to consume salted fish throughout adulthood [11, 16]. In addition, studies of the Chinese population in China reported that multiple food items, including preserved and fermented foods, were significantly associated with NPC during weaning and children exposure before 2 years of age [12]. Furthermore, risk factors such as occupational exposure to smoke and dust [15] and genetic variants [7] were shown to be associated with NPC. In 1994, Lee et al. [10] studied Singaporean Chinese patients with NPC who were younger than 45 years and found an increased risk of NPC in adults who frequently consumed salted food. In 2006, a Singaporean Chinese cohort study found that smoking duration, smoking intensity, age of smoking initiation, and alcohol consumption were strongly associated with an increased risk of other oropharyngeal carcinomas; however, they found that the association of these factors with NPC was rather weak, except for those who had smoked for 40 or more years [17]. This previous cohort study did not specifically focus on the risk factors for NPC in a Singaporean population, and the non-significant finding was likely the result of the small sample size of NPC cases.

Tobacco smoking and alcohol consumption have been widely studied in Southeast Asia [7, 15, 18, 19] and in Western countries such as the United States [20–22] and Italy [23], with no consistent relationships found between lifestyle factors and the risk of NPC. Although many studies have reported inconsistent results, in 2012 the World Health Organization (WHO) reported that tobacco use was an established risk factor for many cancer types, including NPC [24]. However, the relative importance of tobacco exposure in different NPC histologic subtypes remains largely unexplored [25–27], and additional prospective studies are needed. Because of smoking’s modifiable nature and causal relationship in the development of various cancers, it is an important lifestyle risk factor to target. In 2012, WHO in the Western Pacific region reported that China (which is a high-risk area for NPC) had more than 300 million smokers—one-third of the world’s total [28]. In our study, we postulated that given the higher incidence of smoking as countries (e.g., China) become further developed, given the increased accessibility of treatment, given the rapid aging of populations, and given the increasing median age of survival, the burden of cancer (prevalence) will increase.

The nitrosamines in tobacco are known to be active carcinogenic metabolites that cause DNA damage and chronic inflammation in nasopharyngeal mucosa [25]. Organs in direct contact with smoke—the oral cavity, esophagus, and lungs—have high risks of developing cancer. Similarly, the nasopharynx, which is located between the nasal cavity and the larynx, is also directly exposed to tobacco smoke. As tobacco smoking is a modifiable risk factor, indeed, 30% of all cancers reported in the United States could be prevented if smoking were eliminated [29]. Tobacco contains carcinogens that may interact with other exposures, such as alcohol, resulting in an increased risk of cancer [29]. Therefore, smoking cessation is the single most important and effective way to reduce a person’s risk of cancer [29, 30].

In the present study, we examined the lifestyle factors (smoking, alcohol consumption, various salted and preserved food consumption, pre-chewing of food, and weaning practices) involved in the development of NPC in the Singaporean population. Since Singapore is a high-risk area for the development of NPC, studying the risk factors in this population provides more data for future studies of NPC in other Southeast Asian countries. The National Cancer Centre of Singapore (NCCS) is the country’s largest tertiary referral center for NPC patients; as such, NPC patients recruited from there are representative of all Singaporean patients diagnosed with NPC.

## Patients and methods

### Study population

Between 2008 and 2012, 300 patients between 21 and 80 years of age who were Singaporeans or permanent residents of Singapore were recruited from the NCCS. All patients were newly diagnosed with histologically confirmed NPC. The controls were friends of patients, support group volunteers in hospitals and other organizations, members of religious societies, and NCCS staff members. Since Singapore is a small country with a fairly homogenous population, people’s geographic location does not affect demographics and their exposure to the risk factors studied. Controls and patients were recruited simultaneously, and a total of 310 controls were recruited to match the cases. From the databank of 310 controls, one control was selected randomly and matched with each case in terms of age (±5 years), sex, and ethnicity using statistical software. Eligible controls included healthy Singaporeans or permanent residents of Singapore between 21 and 80 years old with no history of cancer and/or cancer-related disease. This study was approved by the Ethical Review Board for research in SingHealth cluster, Singapore. All participants provided informed consent.

### Data collection

Data were collected from patients and controls via face-to-face interviews conducted in the NCCS by trained researchers using a standardized lifestyle questionnaire. This questionnaire was adapted from the International Agency for Research on Cancer (IARC) as part of a joint-collaboration in a wide study of the genetic epidemiology of NPC in Southeast Asia [7]. Information collected included demographics, tobacco smoking, alcohol drinking, dietary habits (consumption frequency of traditional preserved food), and history of weaning (which included history of being breastfed, specific food items consumed, and whether food was pre-chewed). Date of diagnosis and clinical variables were obtained from patients’ clinical records.

### Data analysis

Descriptive statistics were used to summarize the general demographic characteristics of the study population, stratified by cases and controls. The frequency of the food consumed was classified into three categories: never or rarely consumed, consumed at least once a month (monthly basis), and consumed at least once a week (weekly/daily basis). Where appropriate, categorical variables were assessed by the Chi square test or Fisher’s exact test; continuous variables were assessed by *t* tests. The matched-pair method was used to analyze the data [31]. Associations between the risk factors and NPC were examined singly using conditional logistic regression to obtain the crude odds ratio (OR) and its corresponding 95% confidence interval (CI), followed by multivariable analysis, adjusting for covariates and interaction effects if necessary [32]. Adjusted ORs were reported from the multivariate model that was best fit obtained during statistical analysis. Additionally, to assess the possibility of dose–response relationships with risk of NPC, overall trend (*P*-*trend*) for the consumption of the food items was computed. Case–control pairs that had missing values in either case or control were excluded from the analysis. Unconditional logistic regression adjusting for age, sex, and ethnicity was also performed; the results were similar to those observed when conditional logistic regression was used. All statistics presented were obtained by conditional logistic regression. All statistical analyses were performed using STATA version 12.0 software (Stata Corporation, College Station, TX, USA). Two-sided tests with *P* values less than 0.05 were considered statistically significant.

## Results

### Demographics and clinical characteristics of study population

In this study, from the total of 300 patients and 310 controls, only 290 pairs of cases and controls were identified and matched successfully. The remaining 10 cases were unable to be matched and were excluded from this study. The main characteristics of patients and controls are summarized in Table 1. Of the 290 pairs of patients and controls, 236 (81.4%) were men, and 54 (18.6%) were women. The difference of mean age of patients (49.9 years) and controls (48.3 years) was not statistically significant (*P* = 0.069). Most NPC patients were Chinese (94.1%), followed by Malays (5.2%) and Indians (0.7%). Marital status (*P* = 0.519) and the various dialects (*P* = 0.051) were not significantly different between the Chinese patients and controls. However, in this study, the proportion of those with less education was higher among the patients than among the controls, and the difference was statistically significant (*P* < 0.001). Based on WHO histologic classification, 231 patients (79.6%) were categorized as having type III NPC (non-keratinizing undifferentiated carcinoma), 55 (19.0%) were categorized as having type II NPC (non-keratinizing carcinoma), and 2 (0.7%) were categorized as having type I NPC (squamous cell carcinoma) (Table 1).

**Table 1:** Baseline characteristics of 290 pairs of study participants

Variable	Patients	Controls	*P* value^c^
Age (years)			0.069
Mean ± standard deviation	49.9 ± 10.3	48.3 ± 10.8	
Sex			1.000
Men	236 (81.4)	236 (81.4)	
Women	54 (18.6)	54 (18.6)	
Ethnicity			1.000
Chinese	273 (94.1)	273 (94.1)	
Malay	15 (5.2)	15 (5.2)	
Indian	2 (0.7)	2 (0.7)	
Marital status			0.519
Not married	47 (16.3)	60 (20.7)	
Widowed	4 (1.4)	4 (1.4)	
Divorced	11 (3.8)	8 (2.8)	
Currently married	228 (78.6)	218 (75.2)	
Education			<0.001
None	6 (2.1)	2 (0.7)	
Primary	52 (17.9)	11 (3.8)	
Secondary	117 (40.3)	70 (24.1)	
Tertiary and higher	115 (39.7)	207 (71.4)	
Chinese dialect^a^			0.051
Cantonese	46 (16.9)	61 (22.3)	
Hakkas	25 (9.2)	21 (7.7)	
Hokkien	125 (45.8)	96 (35.2)	
Teo Chew	55 (20.2)	60 (22.0)	
Others^b^	22 (8.1)	35 (12.8)	
WHO type of NPC			
I	2 (0.7)	NA	
II	55 (19.0)	NA	
III	231 (79.6)	NA	
Unknown	2 (0.7)	NA	

Except for the data of age, other values are presented as the number of pairs followed by percentages in the parentheses

*NA* not applicable

^a^Among 290 pairs of participants, 273 were Chinese

^b^Other dialects included Hainanese, Fuchow, Hinghwa, HockChia, Peranakan, Shangainese, and Wenzhou

^c^Based on Chi square test, Fisher’s exact test, or *t* test where appropriate; *P* values less than 0.05 were considered statistically significant

### Associations of lifestyle and diet with NPC risk factors

Univariate conditional logistic regression analyses of lifestyle, diet, and weaning are listed in Table 2. Tobacco smoking was significantly associated with the risk of NPC (current smokers: OR = 4.50, 95% CI 2.61–7.78; former smokers: OR = 2.37, 95% CI 1.48–3.79), but the association of alcohol drinking with risk of NPC was not statistically significant (Table 2). Of the food items examined, the participants who consumed salted meat at least once a month was found to have doubled risk of developing NPC compared with those participants who never or rarely consumed salted meat (OR = 2.04, 95% CI 1.18–3.50). In addition to salted meat, salted vegetables consumed at least once a week were also found to significantly associated with an increased risk of developing NPC (OR = 3.70, 95% CI 1.58–8.64) compared with salted vegetables rarely consumed. The trend of increasing risk of NPC was significantly associated with increasing frequency of salted fish, salted meat, and salted vegetable consumption (*P*-*trend* = 0.033, 0.003, and <0.001, respectively).

**Table 2:** Association between lifestyle variables, dietary habits, and weaning practices and the risk of nasopharyngeal carcinoma

Variable	Matched-pair analysis			*P* value^e^
	No. of cases (%)	No. of controls (%)	Crude OR (95% CI)	
Lifestyle				
Tobacco smoking				
Never	146 (50.3)	212 (73.1)	1	
Current	79 (27.2)	33 (11.4)	4.50 (2.61–7.78)	<0.001
Former	65 (22.4)	45 (15.5)	2.37 (1.48–3.79)	<0.001
Alcohol consumption^a^				
No	114 (39.3)	120 (41.4)	1	
Yes	176 (60.7)	170 (58.6)	1.11 (0.77–1.59)	0.581
Dietary^b^				
Salted fish				
Never/rarely	251 (86.6)	264 (92.0)	1	
Monthly	32 (11.0)	20 (7.0)	1.67 (0.93–2.99)	0.087
Weekly/daily	7 (2.4)	3 (1.0)	2.33 (0.60–9.02)	0.220
*P*-*trend*				0.033
Salted meat				
Never/rarely	222 (76.8)	248 (86.4)	1	
Monthly	47 (16.3)	28 (9.8)	2.04 (1.18–3.50)	0.010
Weekly/daily	20 (6.9)	11 (3.8)	2.18 (0.97–4.89)	0.059
*P*-*trend*				0.003
Salted vegetables				
Never/rarely	205 (70.7)	237 (81.7)	1	
Monthly	61 (21.0)	46 (15.9)	1.54 (0.99–2.39)	0.053
Weekly/daily	24 (8.3)	7 (2.4)	3.70 (1.58–8.64)	0.002
*P*-*trend*				<0.001
Smoked fish				
Never/rarely	264 (98.5)	259 (89.9)	1	
Monthly	22 (7.6)	26 (9.0)	0.84 (0.47–1.50)	0.555
Weekly/daily	4 (1.4)	3 (1.0)	1.33 (0.30–5.96)	0.706
*P*-*trend*				0.816
Smoked meat				
Never/rarely	240 (82.8)	231 (79.9)	1	
Monthly	39 (13.5)	51 (17.7)	0.75 (0.47–1.18)	0.214
Weekly/daily	11 (3.8)	7 (2.4)	1.52 (0.55–4.24)	0.419
*P*-*trend*				0.538
Preserved vegetables				
Never/rarely	216 (74.5)	230 (81.0)	1	
Monthly	56 (19.3)	44 (15.5)	1.33 (0.85–2.07)	0.213
Weekly/daily	18 (6.2)	10 (3.5)	1.86 (0.82–4.23)	0.138
*P*-*trend*				0.061
Rusip/Chincalup^c^				
Never/rarely	277 (95.9)	268 (96.8)	1	
Monthly	10 (3.5)	5 (1.8)	1.93 (0.66–5.68)	0.232
Weekly/daily	2 (0.7)	4 (1.4)	0.55 (0.10–3.06)	0.496
*P*-*trend*				0.866
Weaning practices^d^				
Breastfed				
No	70 (24.1)	83 (28.6)	1	
Yes	134 (46.2)	141 (48.6)	1.29 (0.83–2.02)	0.259
Unknown	86 (29.7)	66 (22.8)		
Porridge				
No	4 (1.4)	10 (3.5)	1	
Yes	218 (75.2)	212 (73.1)	2.25 (0.69–7.31)	0.177
Unknown	68 (23.5)	68 (23.5)		
Salted fish				
No	117 (40.3)	164 (56.6)	1	
Yes	59 (20.3)	27 (9.3)	2.21 (1.18–4.16)	0.014
Unknown	114 (39.3)	99 (34.1)		
Salted meat				
No	126 (43.5)	169 (58.3)	1	
Yes	43 (14.8)	17 (5.9)	2.56 (1.18–5.52)	0.017
Unknown	121 (41.7)	104 (35.9)		
Preserved vegetables				
No	121 (41.7)	157 (54.1)	1	
Yes	53 (18.3)	32 (11.0)	2.15 (1.12–4.16)	0.003
Unknown	116 (40.0)	101 (34.8)		
Tin/canned food				
No	104 (35.9)	142 (49.0)	1	
Yes	75 (25.9)	45 (15.5)	1.78 (1.0–3.17)	0.051
Unknown	111 (38.3)	103 (35.5)		
Pre-chewed food				
No	119 (41.0)	154 (53.1)	1	
Yes	51 (17.6)	59 (20.3)	1.68 (1.0–2.85)	0.053
Unknown	120 (41.4)	77 (26.6)		

*OR* odds ratio, *CI* confidence interval

^a^Inclusive of current and past drinkers

^b^Analysis excludes missing values (range, 1–13 missing values)

^c^Traditional food of Bangka people and is made from fermented anchovies

^d^Analysis excludes the cases and controls with unknown data

^e^Based on conditional logistic regression, *P* values less than 0.05 were considered statistically significant

The association between the risk of NPC and the consumption of food items such as salted fish, smoked fish, smoked meat, preserved vegetables, and rusip/chincalup was not found to be statistically significant (Table 2). In addition, the consumption frequencies of smoked fish, smoked meat, preserved vegetables, and rusip/chincalup were similar between patients and controls (*P* = 0.082, 0.538, 0.061, and 0.866, respectively) (Table 1).

Traditional preserved foods, such as salted fish (OR = 2.21, 95% CI 1.18–4.16), salted meat (OR = 2.56, 95% CI 1.18–5.52), and preserved vegetables (OR = 2.15, 95% CI 1.12–4.16), that were consumed during the weaning period were significantly related to an increased NPC risk. The unknown values of weaning information were similar between the patients and controls (Table 2). Other food items consumed during the weaning period, such as breast milk (*P* = 0.259), porridge (*P* = 0.177), and canned food (*P* = 0.051), were not significantly associated with NPC risk. Similarly, no association was found between the practice of pre-chewing food and the risk of NPC (*P* = 0.053).

In the multivariate model, the participants who smoked tobacco and those who had ever smoked and consumed salted vegetables at least once a week were significantly associated with increased risks of NPC (OR = 4.50, 95% CI 2.58–7.86; OR = 2.52, 95% CI 1.54–4.12; and OR = 4.18, 95% CI 1.69–10.38, respectively, after adjusting for education level) (Table 3). No interaction effects were found in the model. Moreover, the food items consumed during the weaning period that were significantly associated with the risk of NPC (salted fish, salted meat, and preserved vegetables) in the univariate analysis were not statistically significant in the multivariate analysis (Table 3).

**Table 3:** Analysis of the cases and the controls by multivariate conditional logistic regression

Variable	Adjusted OR^a^	95% CI	*P* value
Tobacco smoking			
Never	1		
Current	4.50	2.58–7.86	<0.001
Former	2.52	1.54–4.12	<0.001
Salted vegetables			
Never/rarely	1		
Monthly	1.41	0.88–2.26	0.150
Weekly/daily	4.18	1.69–10.38	0.002

*OR* odds ratio, *CI* confidence interval

^a^ Adjusted for educational level of cases and controls

## Discussion

In our study, we found that tobacco smoking (by both current and former smokers) was a statistically significant risk factor for developing NPC. Compared with never smokers, current smokers had four times the risk of developing NPC, and those who had ever smoked had double the risk. Studies conducted in Taiwan, China [5], in Thailand [6, 7], in Wuhan, China [8], in Shanghai, China [14], and in the United States [22] also found that smoking was a risk factor for NPC, although other studies in Singapore [17], Malaysia [33], Serbia [34], and China [35] found the association to be less clear but did suggest that the inhalation of passive smoke during childhood could affect the risk of developing NPC [34]. A recent meta-analysis found that ever smokers had a significantly higher risk of developing NPC than never smokers, but these associations were more relevant to squamous cell carcinoma and less relevant in undifferentiated carcinoma [27]. Since most patients in our study (80.0%) had undifferentiated carcinoma type of NPC, our results seem to suggest that exposure to tobacco smoke is equally relevant to undifferentiated carcinoma and the Singaporean population.

The literature offers several possible explanations regarding the carcinogenic mechanism. Tobacco smoke comes in direct contact with the nasopharynx and, therefore, might have direct action. However, it has also been proposed that tobacco is just one of many risk factors for NPC and may contain Epstein-Barr virus (EBV)-activating substances [26]. It is known that EBV infection is strongly associated with undifferentiated carcinoma, and in high-risk areas 95% of NPC patients have this type of carcinoma [27]. Several epidemiological studies have suggested that high concentrations of volatile nitrosamines are found in tobacco smoke [12], and these compounds were positively associated with cancer in other sites, such as the esophagus [36] and possibly the nasopharynx [12]. With regard to alcohol drinking, our results were similar to those of other studies, finding no strong association between alcohol drinking and NPC risk [4–6, 14, 17].

Additionally, we observed that people who consumed salted vegetables at least once a week had four times the risk of developing NPC compared with those who never or rarely consumed salted vegetables. This observation is consistent with the results of studies conducted in 1994 in Singapore [10], in 1998 in Malaysia [4], and in 2000 [14] and 2010 in China [9], all of which suggested that frequent consumption of salted vegetables was associated with risk of developing NPC. Salted vegetables are widely consumed in Singapore, with 18.3% of the control population in this study being regular consumers. These products are readily available in the supermarket, and people generally consume salted vegetables as part of a meal. The salted vegetables called suan cai is a traditional Chinese pickled cabbage—a unique type of cabbage and mustard greens because of the ingredients used and the method of production [37]. It is usually prepared first by being pressed slowly and fermented, followed by pickling with salt and brine [38]. It is similar to sauerkraut, which is a common food in Central and Eastern Europe [37]. On the other hand, preserved vegetables referred to other types of fermented vegetables or fruits or marinated in mixtures based on soy sauce and soybean paste [38]. Little is known about particular carcinogenic compounds found in salted vegetables that affect the nasopharynx; more research on this relationship is warranted. On univariate analysis, we found, in contrast to previous studies [4, 9, 14, 35], no clear significant association between adult consumption of salted fish and NPC risk. This could be due to the broad decreased consumption of salted fish in populations with high risk of NPC such as Singaporeans [25]. Indeed, since 1980, a markedly decreasing trend in salted fish consumption has been observed in similar high-risk areas such as Hong Kong, China [25, 39]. Not surprisingly, the Singaporean population, the majority of which is of Chinese ethnicity, has also changed their diet to a more Westernized and international one, replacing the traditional Chinese diet. Furthermore, the harms associated with the frequent consumption of salted fish, although not salted vegetables, was widely publicized in Singapore [40].

Our findings may have several limitations, particularly those inherent to retrospective case–control studies. First, case–control studies are vulnerable to recall bias when information can be collected only through the recall of study participants—this was the case for our study. We tried minimizing recall bias by asking confirmation questions and, on dietary questions, having several options in terms of consumption frequency. Second, our data collection was limited by the questions in the questionnaire asked directly about the study participants, particularly with regards to lifestyle, dietary information, and weaning practices. Data on weaning are ideally obtained from the mother of the study participant, rather than the participant himself or herself. But given that cancer is a late-onset disease, the mother may no longer be alive or may not remember this information, so the information was self-reported by the participant where possible. Notably, the amount of missing data on weaning was similar across cases and controls. Nonetheless, none of the weaning variables was conclusive, and these variables were not included in the multivariate model. Our results must be interpreted in the context of these constraints. Third, we recruited controls from our staff, friends of patients, and various organizations (both hospital and community). We acknowledge that there could be selection bias in our controls because controls were people who understood the importance of such studies and were willing to participate for the benefit of society. Also, they were more likely to have higher education. In Singapore, the general population’s participation rate in scientific studies is nearly low, and often significant effort is needed to recruit enough controls. We tried minimizing selection bias by recruiting controls from many different organizations; at the same time we recruited cases of approximately the same age, sex, and ethnicity. Because of the fairly homogenous population in Singapore, recruiting controls from various sources did not create significant biases. Fourth, we adjusted for educational level when analyzing the risk factors of NPC. Fifth, interviewer bias was one additional concern because knowledge and understanding of the risk factors by the interviewers and study participants may cause skewed results. When the study participants and interviewers are aware of the risk factors, the patients are more likely than the controls to pay greater attention to risk factor information during data collection. Therefore, we reduced the risk of interviewer bias by having only two trained interviewers who employed a standardized interview technique, using the same lifestyle questionnaire for both patients and controls. Sixth and finally, the lack of information regarding the EBV status of each participant was another potential limitation. We are aware that EBV status could be a main risk factor for the development of NPC and could potentially have influenced the results of our study. However, EBV is fairly common in Singapore, and seroconversion occurs at an early age, with a rate of EBV seropositivity of 40%–60% in children younger than 5 years.

## Conclusions

In summary, we found that certain modifiable risk factors, namely smoking and frequent consumption of salted vegetables, contribute significantly to the development of NPC. This finding is important because understanding the risk factors of NPC could potentially lead to changes in cancer prevention campaigns. The burden of NPC is a major public health concern in Singapore, and by knowing that the risk factors are modifiable, public health programs might be better able in the future to reduce the burden of NPC by allocating resources and formulating population strategies accordingly. Although it may take a long time to adopt the cancer prevention strategies that could reduce the incidence of NPC, our findings could help tailor such important strategies, which hold significant future promise.

## Abbreviations

ASR: age-standardized rate
CI: confidence interval
EBV: Epstein-Barr virus
IARC: International Agency Research on Cancer
NA: not applicable
NPC: nasopharyngeal carcinoma
OR: odds ratio
SD: standard deviation
WHO: World Health Organization
